# Disorder-mediated crowd control in an active matter system

**DOI:** 10.1038/ncomms10907

**Published:** 2016-03-09

**Authors:** Erçağ Pinçe, Sabareesh K. P. Velu, Agnese Callegari, Parviz Elahi, Sylvain Gigan, Giovanni Volpe, Giorgio Volpe

**Affiliations:** 1 Department of Physics, Bilkent University, Çankaya, 06800 Ankara, Turkey; 2 Laboratoire Kastler Brossel, Université Pierre et Marie Curie, École Normale Supérieure, CNRS, College de France, 24 rue Lhomond, 75005 Paris, France; 3 UNAM-National Nanotechnology Research Center, Bilkent University, Çankaya, 06800 Ankara, Turkey; 4 Department of Chemistry, University College London, 20 Gordon Street, London WC1H 0AJ, UK

## Abstract

Living active matter systems such as bacterial colonies, schools of fish and human crowds, display a wealth of emerging collective and dynamic behaviours as a result of far-from-equilibrium interactions. The dynamics of these systems are better understood and controlled considering their interaction with the environment, which for realistic systems is often highly heterogeneous and disordered. Here, we demonstrate that the presence of spatial disorder can alter the long-term dynamics in a colloidal active matter system, making it switch between gathering and dispersal of individuals. At equilibrium, colloidal particles always gather at the bottom of any attractive potential; however, under non-equilibrium driving forces in a bacterial bath, the colloids disperse if disorder is added to the potential. The depth of the local roughness in the environment regulates the transition between gathering and dispersal of individuals in the active matter system, thus inspiring novel routes for controlling emerging behaviours far from equilibrium.

## Abstract (web summary)

Many living systems, such as bacterial colonies, exhibit collective and dynamic behaviours that are sensitive to the change in environmental conditions. Here, the authors show that a colloidal active matter system switches between gathering and dispersal of individuals in response to a disordered potential.

## Graphical abstract

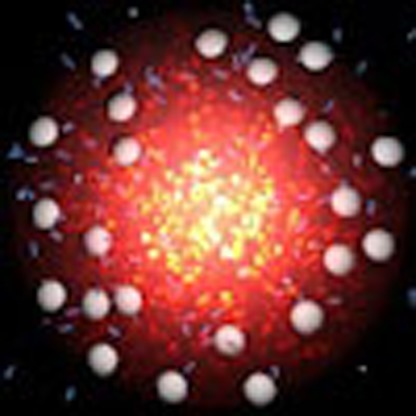

The spatial organization of individuals plays a crucial role in the growth and evolution of complex systems. Their gathering and dispersal are critical in phenomena as diverse as the genesis of planetary systems^1^, the organization of ecosystems and human settlements^2^, the growth of bacterial colonies and biofilms^3^,^4^,^5^,^6^, the self-organization of active matter systems^7^,^8^ and the assembly of macromolecular complexes at the nanoscale^9^,^10^. In systems close to thermal equilibrium, this is the case in the formation and melting of crystals^11^. For systems that are far from equilibrium, such as living active matter, these dynamics become much less intuitive and can sensitively depend on environmental factors^12^,^13^. Typical environments for natural active matter systems can indeed be highly heterogeneous, and, as recent theoretical work has shown^14^,^15^, the presence of spatial disorder can significantly influence the motility of active particles, thus leading an active system to different long-term behaviours. Despite these theoretical insights, the difficulty of experimentally exploring complex environments in a controllable way has held back the study of these dynamics in active matter systems.

Here, we explore the long-term spatial organization of a population of colloids in an active bath under diverse environmental conditions where a controllable degree of disorder is introduced with optical potentials^16^. The colloidal particles are driven far from thermal equilibrium by an active bath of motile *Escherichia coli* (*E. coli*) bacteria (Methods section)^17^, which are self-propelling microorganisms whose motion proceeds as an alternation of running and tumbling events^18^; because of random collisions with the bacteria in the solution, the colloids are driven far from equilibrium, and, in a homogenous environment, their motion features a crossover at a characteristic time in the order of a few seconds from ballistic motion at short times to enhanced diffusion at long times with an effective diffusion coefficient that is higher for higher concentrations of bacteria^17^. The colloids in the active bath thus effectively behave like active particles^19^,^20^. Differently from a system at equilibrium, our results show that the presence of spatial disorder in an external attractive potential alters the long-term dynamics of the colloidal active matter system: in particular, the depth of the local roughness in the environment regulates the transition between individuals gathering in and dispersing from the attractive potential, thus inspiring novel routes for controlling emerging behaviours far from equilibrium.

## Results

### Dynamics in smooth potentials

**Figure 1:**
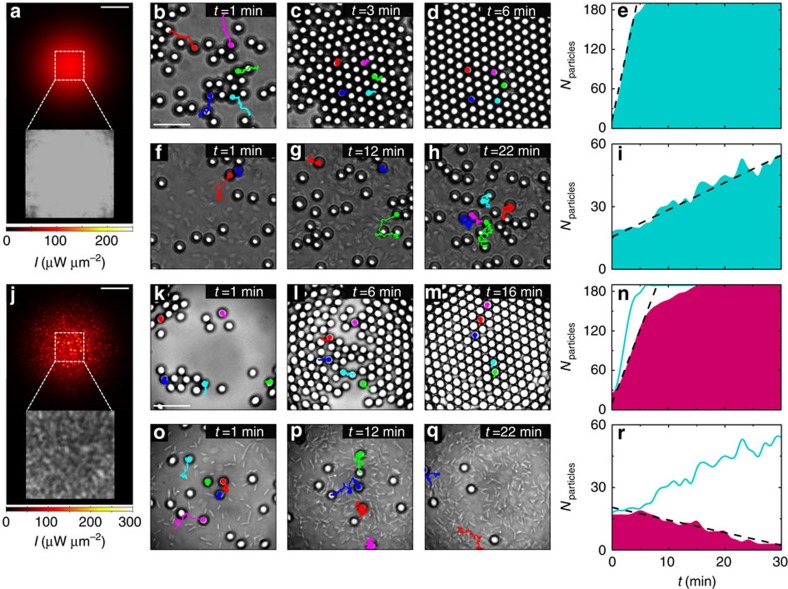
Gathering and dispersal of colloids in an active bath. In a smooth attractive optical potential generated by a Gaussian beam (*λ*=976 nm, *w*_0_=47.8±0.2 μm and *P*=100 mW) (**a**) the (**b**–**d**,**f**–**h**) time sequences show colloids (silica microspheres, *d*=4.99±0.22 μm) gathering at the centre of the illuminated area (corresponding to the dashed square in (**a**,**j**)) in a thermal bath and in an active bath of *E. coli* bacteria, respectively. When disorder is added to this potential with a speckle pattern (**j**) the (**k**–**m**,**o**–**q**) time sequences show that colloids still gather at the centre in a thermal bath, but they are expelled from it in an active bath. The solid lines in the sequences show particles trajectories over 1 min before each snapshot; in each time sequence, trajectories with the same colour correspond to the same particle. The concentration of the bacteria as a function of time is similar in both sequences (**f**–**h**,**o**–**q**) in particular, it starts at a concentration *c*_0_=0.014±0.001 cells per μm^2^ and it reaches a plateau ∼3.5 times this value as time passes. Sample experimental intensity distributions are shown in the insets in **a** and **j**. The shaded areas in **e**,**i**,**n** and **r** show the time evolution of the colloidal population for the four previous cases respectively. The dashed lines are linear fits whose slopes give the initial rate of particle gathering or dispersal. To directly compare smooth and rough potentials, the time evolutions of **e** and **i** are also shown as solid lines in **n** and **r** respectively. The scale bars correspond to 60 μm in **a** and **j** and to 20 μm in **b** and **k**.

To set the stage, we first consider the simple configuration where we illuminate the colloidal particles (silica microspheres, diameter *d*=4.99±0.22 μm) in an equilibrium thermal bath, for example, in absence of bacteria, with a defocused Gaussian beam (wavelength *λ*=976 nm, waist *w*_0_=47.8±0.2 μm, power *P*=100 mW) whose intensity profile is reproduced in Fig. 1a (Methods section and Supplementary Fig. 1). We tracked the motion of the colloids by digital video microscopy^21^; their trajectories over 1 min preceding each snapshot are represented by solid lines in the time-lapse sequence in Fig. 1b–d. The Gaussian beam generates a shallow smooth optical potential (Supplementary Fig. 1) that attracts the colloids towards the maximum of intensity in the centre of the illuminated area at an initial rate of 40.2 particles per minute (Fig. 1e); convection or thermophoresis are negligible for the wavelength, power and chamber geometry used in our experiments (Supplementary Note 1). In absence of non-equilibrium driving forces (the bacterial bath), the particles form a crystal-like packed ordered structure within a few minutes from the activation of the potential (Fig. 1d)^11^.

As the time-lapse sequence in Fig. 1f–h shows, the colloids gather at the bottom of the same attractive potential also in an active bacterial bath, albeit without forming a crystal-like structure (Fig. 1h). On average the particles drift towards the maximum of intensity, even though the stochastic nature of the active bath occasionally drives the colloids away from it, as demonstrated by their trajectories in Fig. 1f–h; the overall result is that the colloidal population in the central region increases over time: within the first 30 min from the activation of the potential, the population increases from *N*_particles_≈20 to *N*_particles_≈55 as new individuals gather at a rate of 1.3 particles per minute (Fig. 1i). The effective radial drift, which is negative, also confirms the gathering of particles at the bottom of the potential (Supplementary Table 1 and Supplementary Note 2). Since we start from a disperse solution of colloids and bacteria (Methods section), we do not observe phase transitions^13^ or the formation of active crystals^7^,^8^ within the time frame of our experiment.

### Dynamics in rough potentials

To test the effect of spatial disorder on the active matter system, we now make the potential rough by generating an optical speckle pattern by mode-mixing a coherent laser beam in a multimode optical fibre (Fig. 1j; Methods section and Supplementary Fig. 1). Speckle patterns form rough, disordered optical potentials characterized by wells whose average width is given by diffraction (the average grain size, here *w*_s_=4.87±0.70 μm)^16^. Moreover, the well depths are exponentially distributed^16^, similar to the potentials found in many natural phenomena such as in the anomalous diffusion of molecules within living cells^22^. Since the fibre imposes a Gaussian envelope to the speckle pattern^23^, this random optical potential has a global minimum, which, just as the smooth Gaussian profile, attracts the colloids towards the centre of the illuminated area (Supplementary Fig. 1). In absence of bacteria, in fact, the colloids gather at the centre of the illuminated area and eventually form a crystal-like ordered structure as shown in the time-lapse sequence in Fig. 1k–m. Compared with the sequence in Fig. 1b–d, this process happens at a slower rate of 22.4 particles per minute (Fig. 1n), because the colloids are metastably trapped by the high-intensity grains of the static speckle pattern and undergo a process of transient subdiffusion (Methods section)^16^, as confirmed by the fact that the 1-min trajectories in Fig. 1k are much more confined than those in Fig. 1b.

In the active bath, because of the attractive nature of the optical potential, one would still expect to observe the colloids gather at the centre of the illuminated area as in all previous cases. Yet, the time-lapse sequence presented in Fig. 1o–q shows the opposite behaviour where the colloids are expelled from the central illuminated area—this is our central experimental result. The particle trajectories in Fig. 1o–q clearly show that on average the particles move outwards, even though, as previously noted, there are instances of particles moving in the opposite direction as a consequence of the stochastic nature of this process. The corresponding colloidal population, which at the beginning (at the time of activation of the potential) is similar in number to that of Fig. 1f (*N*_particles_≈20), decreases to virtually no particle after 30 min at a rate of 0.6 particles per minute (Fig. 1r). The effective radial drift is positive and, thus, also confirms the dispersal of particles away from the illuminated area (Supplementary Table 1 and Supplementary Note 2). These results clearly indicate that, while in absence of bacteria the colloids always gather at the bottom of any attractive potential and eventually form a crystal-like ordered structure^11^, under the non-equilibrium driving forces introduced by the bacterial bath the colloids disperse if spatial disorder is added to the attractive potential. In the following, we explain these observations as a consequence of the presence of two sources of heterogeneity in the system: the first is the gradient in the local concentration of bacteria, and the second is the degree of local roughness of the attractive potential.

### Underlying mechanism

**Figure 2:**
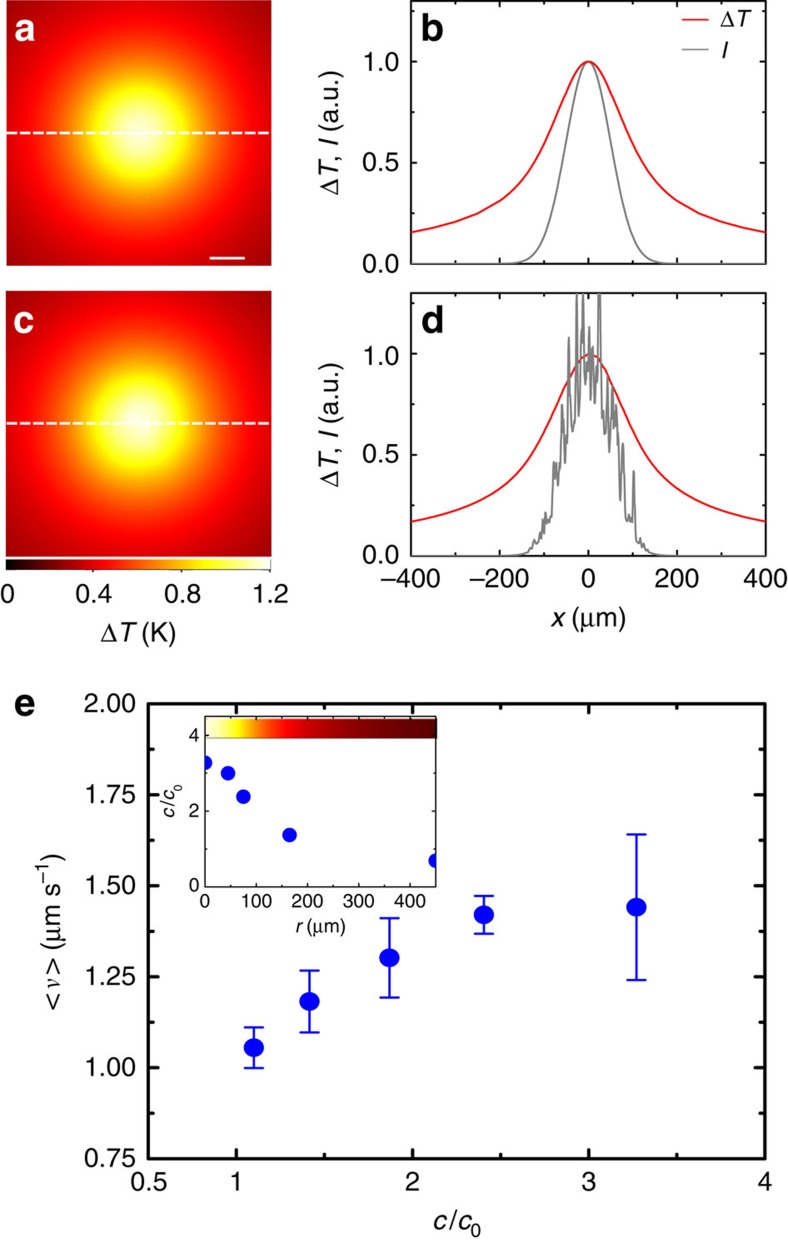
Colloidal average velocity in the temperature-induced gradient of bacteria. (**a**) Calculated temperature gradient Δ*T* near the surface due to light absorption in the motility buffer at *λ*=976 nm for a Gaussian illumination. (**b**) Crosscuts of Δ*T* (red line) and of the Gaussian intensity profile *I* (grey line) along the dashed line in **a**. The scale bar corresponds to 40 μm. (**c**,**d**) Same as (**a**,**b**) for a disordered speckle illumination with a Gaussian envelope. In both cases, the temperature gradient is smooth and is mainly determined by the Gaussian envelope of the intensity distribution, despite the presence of local roughness in the speckle intensity. (**e**) *E. coli* bacteria are attracted towards warmer areas and their radial concentration *c* increases as a function of the local heating (inset), so that the average velocity *v* of the colloids, which depends on the concentration of bacteria, fades radially when moving away from the central illuminated area. *c*_0_ is the concentration of bacteria before the activation of the optical potential and it is homogeneous in space. The error bars represent one s.d. around the average values. The colour bar in the inset shows the temperature variation as a function of position.

To understand why the addition of spatial disorder leads the active system to this counterintuitive long-term response, we need to explore the underlying dynamics behind the behaviour of the bacteria, which are the driving force that takes the system out of equilibrium and represent the first source of heterogeneity in the system. As it can be directly appreciated in the time-lapse sequences in Fig. 1f–h and in Fig. 1o–q, the motile bacteria rapidly accumulate in the illuminated area following the activation of the optical potential. For the power levels we employ, the optical forces acting on the bacteria are negligible (Methods section), whereas absorption of near-infrared light from the motility buffer generates a shallow temperature gradient (Δ*T*=1.3±0.3 K above room temperature; Supplementary Fig. 2 and Supplementary Note 3) that attracts the bacteria towards warmer regions^6^,^24^,^25^,^26^. To visualize the temperature profile, we calculated the steady-state temperature gradient for both smooth (Fig. 2a,b) and rough potentials (Fig. 2c,d). The calculated temperature increase (Δ*T*≈1.2 K above room temperature, Supplementary Note 3) agrees with the measured value and is similar for both potentials since it is mainly determined by the Gaussian envelope of the intensity profile and shows little sensitivity to the local roughness in the speckle case. As a consequence of their accumulation in the illuminated area, the bacteria form a concentration gradient that fades radially towards colder regions^6^,^24^ so that the average velocity *v* of the colloids in the active bath also fades radially as a function of the concentration of bacteria (Fig. 2e)^17^. For both smooth and rough potentials, this creates an outward radial drift that tends to transport the colloids from regions of higher bacterial concentration (higher velocity) to regions of lower concentration (lower velocity), that is, away from the illuminated area. This is in agreement with the theoretical expectation that the density of active particles whose velocity is position-dependent scales with their velocity^27^,^28^. However, only when the colloidal dynamics are slowed down by the transient subdiffusion due to the disorder in the random optical potential—the second source of heterogeneity in the system—this outward drift of colloids outbalances their inward drift due to the attractive nature of the envelope of the optical potentials.

In the bacterial bath, two competing effects influence the dynamics of the colloids: on the one hand, the attractive optical potentials induce a drift towards the centre of the illuminated area for both time-lapse sequences in Fig. 1f–h and in Fig. 1o–q; on the other hand, the temperature-induced gradient of bacteria determines an opposite drift that tends to drive the colloids out of the illuminated area. On average, a single active particle would, in principle, settle where these two counteracting effects balance each other out; this will happen for both cases of smooth and rough potentials, even though at different positions. When multiple particles are present, however, crowding effects due to steric interactions come into play. In the smooth attractive potential (Fig. 1f–h), the particles drifting towards the centre of the illuminated area create a steric confinement that limits the capability of the particles already in the central region to escape from the potential well; hence the overall accumulation. In the rough attractive potential (Fig. 1o–q) instead, the local potential traps introduced by the disorder prevent the formation of a similar steric confinement by significantly slowing down the advancement of the outer, less-active particles towards the bottom of the potential well; hence the overall dispersal. Furthermore, once the particles are expelled, the same roughness created by the local traps is what prevents the particles to re-accumulate at the bottom of the potential well.

**Figure 3:**
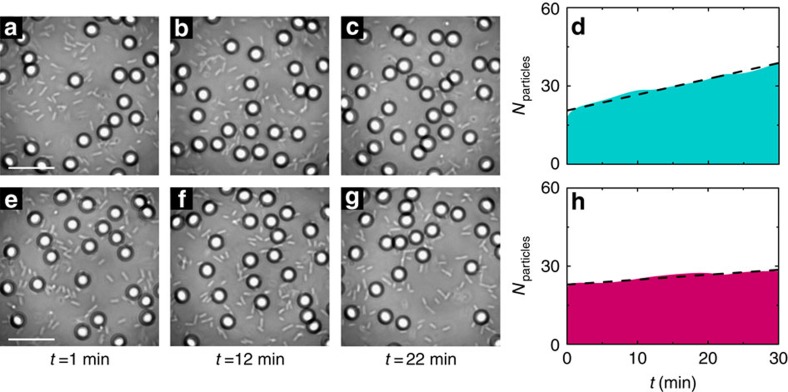
Colloidal dynamics in an active bath at 785 nm. The (**a**–**c**,**e**–**g**) time sequences show the gathering of colloids (silica microspheres, *d*=4.99±0.22 μm) at the centre of the illuminated area in an active bath of *E. coli* bacteria for smooth and rough optical potentials generated by a laser at wavelength *λ*=785 nm (*P*=100 m*W*, *w*_0_=49.9±0.2 μm). The average speckle grain size in **e**–**g** is *w*_s_=4.38±0.50 μm. Because water absorption is about 20 times lower at *λ*=785 nm compared with water absorption at *λ*=976 nm, heating effects are negligible and the gradient of bacteria that can drive the expulsion of colloids from the illuminated area does not form. This is in contrast to Fig. 1f–h,o–q at *λ*=976 nm where bacteria are accumulating at the centre of the illuminated area and gathering of colloids in the active bath is observed only for a smooth potential (Fig. 1f–h), but not for a rough potential (Fig. 1o–q). The scale bars correspond to 20 μm. The shaded areas in **d** and **h** show the time evolution of the colloidal population for the two previous cases, respectively. The dashed lines are linear fits whose slopes give the rate of particle gathering, which is (**d**) 0.6 and (**h**) 0.2 particles per minute, respectively. Compared with the sequence in **a**–**c** the gathering of colloids in **e**–**g** is slowed down by the high-intensity grains of the static speckle pattern where the colloids are metastably trapped.

These conclusions are corroborated by a set of experiments performed with a laser at *λ*=785 nm where water absorption is about 20 times lower than at *λ*=976 nm and heating effects are thus negligible (Δ*T*≈0 K) (Supplementary Fig. 2 and Supplementary Note 3); in this case, we did not observe either accumulation of bacteria or dispersal of colloids, as shown in Fig. 3.

**Figure 4:**
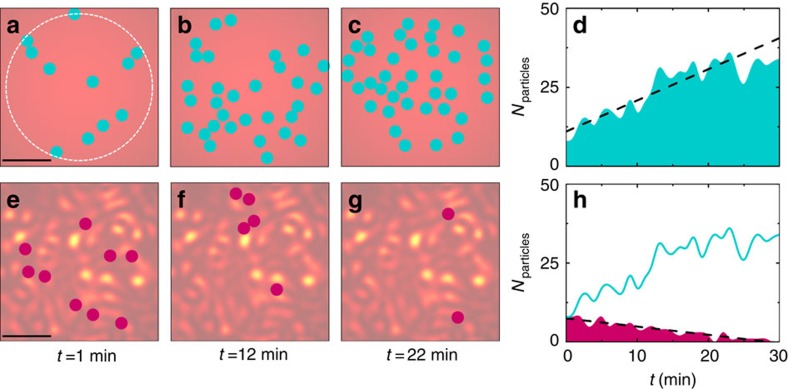
Numerical simulations. The (**a**–**c**,**e**–**g**) time sequences show that active particles gather in a smooth Gaussian potential, while they disperse in a rough spatially disordered potential. The particles move with a position-dependent velocity *v*(*r*) that is constant within the dashed circle in **a** and then fades gradually to zero when radially moving away from it. These simulations are in very good agreement with the experimental time sequences reported in Fig. 1f–h,o–q, respectively. The scale bars correspond to 20 μm. Sample intensity distributions are shown in the background for the two time sequences. The shaded areas in **d** and **h**, respectively, show the time evolution of the active particles for the two previous cases. The dashed lines are linear fits whose slopes give the initial rate of particle gathering or dispersal. To directly compare smooth and rough potentials, the time evolution of **d** is also shown as a solid line in **h**. The simulation parameters are chosen to closely mimic the corresponding experimental values.

To test the generality of these results for a generic active matter system beyond the specific implementation of our experimental settings, we consider a minimalistic numerical model where the colloids are substituted by active particles whose average velocity *v* is position-dependent to account for the temperature-induced gradient of bacteria (Methods section). This is a realistic scenario both for living and artificial active matter, for example, bacteria adjust their propulsion in response to chemical gradients of food or toxins, and microswimmers in response to gradients in their energy source^13^. Figure 4 shows that, also in simulation, the long-term behaviour of a population of active particles with a position-dependent velocity *v* depends on the underlying potential in quantitative agreement with our experimental results in Fig. 1: in a smooth Gaussian potential (Fig. 4a–c), the particles gather at its minimum at a rate of 0.88 particles per minute (Fig. 4d), while in a disordered potential with a Gaussian envelope (Fig. 4e–g) the particles are expelled from the central region at a rate of 0.26 particles per minute (Fig. 4h), despite the presence of attractive forces pushing them inwards.

### Transition from gathering to dispersal

**Figure 5:**
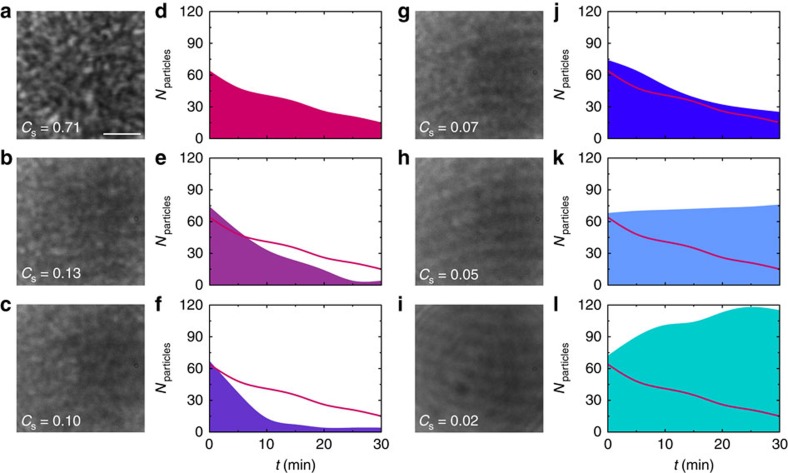
Controlled transition between gathering and dispersal of colloids in an active bath. (**a**–**c**,**g**–**i**) As the local roughness of the laser beam is continuously decreased from (**a**) a high-contrast speckle *C*_s_=0.71 to (**i**) an almost Gaussian distribution with very low-speckle contrast *C*_s_=0.02, the time evolution of the colloidal population in the active bath (**d**–**f**,**j**–**l**) show a non-monotone transition from (**d**) dispersal to (**l**) gathering of individuals in the central illuminated area. To directly compare all different cases, the time evolution in **d** is also shown as a solid line in the other time evolutions. The corresponding snapshots at *t*=30 min of the distribution of colloids are shown in Supplementary Fig. 3. The scale bar corresponds to 20 μm.

So far we have identified two long-term behaviours for the active system under different underlying potentials, that is, the gathering and dispersal of active particles. Figure 5 and Supplementary Fig. 3 show that the transition between such opposite responses can be controlled by regulating the average depth of the local roughness in the potential. To decrease the potential depth in a controllable way, we generate time-varying speckle patterns with different decorrelation times 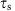 (Methods section and Supplementary Fig. 1): in this way, the effective potential is the time average of the potentials generated by all the *M* uncorrelated speckle patterns within the characteristic timescale of the colloids' motion over the speckle patterns (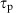
≈124 ms)^16^. The change in local potential depth can then be measured by the speckle contrast 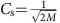, where the factor 2 accounts for the two possible polarizations of light and 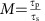 for 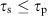 and *M*=1 otherwise^29^. This allowed us to dynamically shift from a potential with maximum contrast when the speckle is static (*C*_s_=0.71, 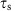
=∞, Fig. 5a) to a case where the speckle is decorrelating extremely fast so that any roughness is averaged out in time and the potential is essentially Gaussian (*C*_s_≈0, 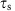
=0.08 ms, Fig. 5i). The evolution of the colloidal populations is shown in Fig. 5 for the various cases. As expected, the two extreme cases (Fig. 5a,i) closely resemble the results of Fig. 1, and we respectively observe dispersal and gathering of colloids. For the intermediate cases, we observe a continuous transition between these two behaviours going through a case where the colloidal population is stable in time (*C*_s_=0.05, 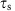
=0.7 ms, Fig. 5h) when the two competing processes, that is, the gathering and the dispersal of colloids are balancing each other. Interestingly, this transition is non-monotone: when we first reduce the contrast (*C*_s_=0.13, 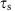
=4.2 ms, Fig. 5b and *C*_s_=0.1, 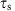
=2.5 ms, Fig. 5c), the dispersal of colloids becomes even faster before starting to slow down for lower values of *C*_s_ (*C*_s_=0.07, 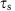
=1.4 ms, Fig. 5g). Our interpretation for this behaviour is that initially, when the contrast of the speckle lowers and the average potential depth starts decreasing, the bacteria can push the colloids out of the local potential wells created by the speckle more easily, thus accelerating the rate of expulsion of the particles from the illuminated area.

**Figure 6:**
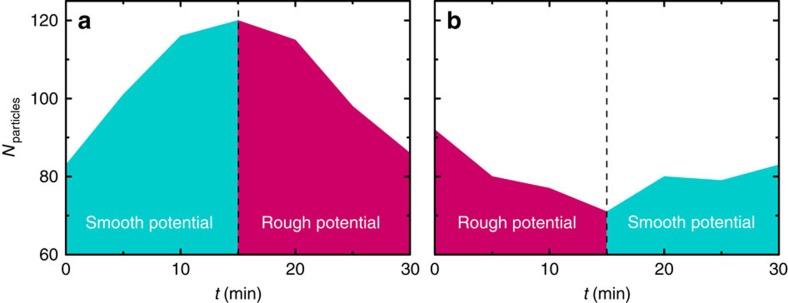
Dynamic switching between gathering and dispersal of colloids in an active bath. By dynamically controlling the roughness of the potential, it is possible to make the active system shift in real time between the two opposite behaviours in Fig. 5d,l. (**a**) The colloids first gather in the illuminated area under a smooth Gaussian potential, while they start to disperse after the first 15 min when the potential is switched to a disordered one. (**b**) The opposite situation is considered where the colloids, after dispersing for the first 15 min in a disordered potential, start gathering again in the illuminated area when the potential is switched to a smooth Gaussian one.

Finally, Fig. 6 shows that real-time control of the dynamics of the active system is achievable by changing the statistical properties of the potential. By switching between smooth and disordered Gaussian potentials and vice versa, the evolution of the colloidal population in time can be modulated and switched between opposite behaviours: in Fig. 6a, the colloids gather in the central area in the first 15 min under smooth Gaussian illumination, and start dispersing as soon as the potential is switched to a disordered one; in Fig. 6b instead the colloids that are being expelled from the disordered attractive potential in the first 15 min restart accumulating as soon as the potential is switched to a smooth one.

## Discussion

Our results demonstrate the critical role played by spatial disorder and environmental heterogeneity in determining the long-term behaviour of active matter systems as a result of non-equilibrium driving forces. The interplay between active particles and the features of the underlying potential where they move can lead to a transition between two long-term opposite behaviours, that is, the gathering and the dispersal of individuals from a common region. Moreover, we have shown that these behaviours can be dynamically controlled by changing the statistical properties of the underlying potential in real time. In particular, we attribute our observations to the interplay among three ingredients, that is, multiple active particles with a position-dependent velocity (which in our experiment is generated by a gradient in bacterial concentration), an attractive potential, and a controllable degree of roughness in the potential, which allows a transition from the gathering of individuals in it to their dispersal. This effect can be explained as a combination of single-particle dynamics and steric collective interactions. While the single-particle dynamics are governed by an intrinsically out-of-equilibrium component associated to the drift in the gradient of the particle velocity, the collective interactions are not specific to active matter systems and they can also be observed for systems at equilibrium (for example, in the crystallization of colloids in a thermal bath in an attractive potential as in Fig. 1b–d, k–m). Similar dynamics can determine the growth, health and survival of living matter systems such as bacterial colonies and biofilms where dispersal and aggregation of individuals play a central role in shaping the time evolution of the population^3^,^5^,^6^. In the study of active matter systems, other interesting phenomena can also emerge as a consequence of the individual or collective interaction of active particles with a disordered environment, such as their spontaneous trapping by disorder^14^ or the emergence of other large-scale collective behaviours due to aligning interactions^30^. Beyond these fundamental interests, these results are relevant to engineer autonomous agents interacting with realistic (complex and crowded) surroundings, for example, artificial microswimmers capable of localizing, picking up and delivering nanoscopic cargos in catalysis, bioremediation, chemical sensing and drug delivery^31^.

## Methods

### Bacteria culture and preparation

Motile *E. coli* were cultured from the wild-type strain RP437 (*E. coli* Genetic Stock Center, Yale University). The bacteria were grown overnight at 32.5 °C in tryptone broth containing 1% tryptone. Once the culture saturated, it was diluted 1:100 into fresh growth medium and incubated again for 4 h at 32.5 °C while mildly shaken at 180 r.p.m. until the culture reached its mid-log phase (OD 600∼0.40). Finally, 5 ml of this dilution was centrifuged at 2,000 r.p.m. at room temperature for 10 min: the resulting precipitated bacterial pellets were then gently collected and resuspended in 5 ml of motility buffer containing 10 mM monobasic potassium phosphate (KH_2_PO_4_), 0.1 mM EDTA (pH 7.0), 10 mM Dextrose (C_6_H_12_O_6_) and 0.002% of Tween 20. This process was repeated three times for replacing the growth medium with motility buffer and halt bacterial growth completely.

### Preparation of the solution of colloids

Diluted solutions of colloids in a thermal bath were prepared by adding 10 μl of monodisperse silica particles (Microparticles GmbH, diameter *d*=4.99±0.22 μm, volume fraction 0.025) to 990 μl of motility buffer. Diluted solutions of colloids in an active bath were instead prepared by adding 10 μl of monodisperse silica particles to 990 μl of motility buffer containing cultured *E. coli* bacteria.

### Experimental set-up and optical potentials

All the experiments are performed on a homemade inverted microscope that is adapted to project both smooth and disordered optical potentials in the sample chamber^23^,^32^, as schematically shown in Supplementary Fig. 1. Smooth Gaussian optical potentials (beam waist *w*_0_=47.8±0.2 μm) are generated by focusing a Gaussian laser beam (*λ*=976 nm, maximum output power *P*=600 m*W*) with a planoconvex lens (*f*=50 mm) onto the sample chamber (Supplementary Fig. 1a). Wavelength and power (*P*=100 m*W*) were chosen to generate a small increase in the temperature of the motility buffer without damaging the bacteria. Optical potentials with different degrees of disorder are generated by coupling the laser beam into a multimode optical fibre (core diameter 105 μm, numerical aperture (NA)=0.22, 51-m long) using a planoconvex lens of short focal distance (*f*=25.4 mm), as shown in Supplementary Fig. 1b. The typical output field, known as speckle, has a random appearance with a Gaussian envelope (beam waist *w*_0_=49.9±0.2 μm) since it is the result of the interference of a large number of optical waves with random phases, corresponding to different eigenmodes of the fibre. In our experiments at λ=976 nm, the average speckle grain size is *w*_s_=4.87±0.70 μm. The fibre is attached to a mechanical oscillator whose vibration frequency can be modulated to change the roughness of the optical potential^16^: when the oscillator is off, the speckle is static in time (decorrelation time 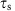
=∞, Supplementary Fig. 1c); otherwise, the frequency of the oscillation can be increased in a controlled manner to have a speckle that decorrelates faster and faster until any roughness is averaged out and the potential is a smooth Gaussian (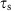
=0.08 ms, Supplementary Fig. 1c). By controlling the speckle decorrelation time between these two extremes, the average depth of the local roughness in the potential can also be controlled. It is worth noting that, for the smooth potential, we obtained qualitatively similar results, that is, gathering of colloids, with both versions of the set-up. The fibre output end is connected to a flat-terminated adapter (Thorlabs, SM1SMA) that constitutes the upper wall of the sample chamber containing the solutions of particles and bacteria; the distance between the top and the bottom of the chamber is *l*≈100 μm. In both versions of the set-up, the particles are tracked by digital video microscopy using the image projected by a microscope objective (× 20, NA=0.5) on a monochrome charge-coupled device (CCD) camera with an acquisition rate in the range of 5–21.4 f.p.s. (ref. 21). Optical scattering forces push the particles in the direction of light propagation towards the lower wall of the sample chamber, so that they effectively confine the particles in a quasi-two-dimensional (2D) space. The incoherent illumination for the tracking is provided by a white-light lamp either directly projected onto the sample (Supplementary Fig. 1a) or coupled into the optical fibre (Supplementary Fig. 1b) using a dichroic mirror (Thorlabs, DMLP605). The typical duration of an experiment is ∼60 min before bacteria motility starts to decrease because of lack of oxygen and nutrients. Supplementary Fig. 1d shows examples of calculated optical potentials for the colloidal particles corresponding to the level of power used in the experiments for both Gaussian and random illumination^23^,^33^. Due to their Gaussian envelope, both potentials show a global minimum at their centre several times deeper than the characteristic thermal energy (≈18*k*_B_*T*). The potential corresponding to random illumination also presents several local minima on its Gaussian envelope that are deep enough (≈4*k*_B_*T* on average for a static speckle) to metastably trap the colloids in the high-intensity grains of the speckle. For the bacteria (for the same levels of power), the global minimum of the Gaussian envelope in the optical potentials is in the order of ≈0.05*k*_B_*T* and the local minima on the Gaussian envelope of the rough potential are in the order of ≈0.3*k*_B_*T*. Both global and local minima are significantly smaller than the thermal energy, thus optical forces on bacteria can be safely neglected.

### Numerical model

We consider a numerical model where the colloids in the active bath are represented by self-propelled hard spheres of radius *R* that move responding to the following set of Langevin equations^34^:




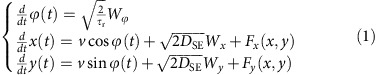




where [*x*(*t*), *y*(*t*)], 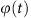, *v*, 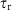, *D*_SE_, *γ*=6*πνR* are, respectively, the active particle's position, orientation, velocity, rotational diffusion time, Stokes-Einstein diffusion coefficient and friction coefficient; *ν* is the viscosity of the surrounding medium; and 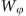 *W*_*x*_ and *W*_*y*_ are independent white noise processes^34^. Therefore, to model the effect of the bacteria on the motion of the colloids, in addition to Brownian motion, the spheres move with a radially-dependent velocity *v*(*r*) in a direction that changes randomly on a timescale determined by an effective rotational diffusion 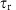
34. The position-dependent velocity of the active particles accounts for the fact that, in the experiment, the bacterial concentration and, thus, the velocity of the colloids are position-dependent as a consequence of the temperature gradient. Then, *v*(*r*) is chosen to reproduce the experimental time dynamics of gathering and dispersal shown in Fig. 1f–h,o–q: *v*(*r*) is constant within the dashed circle in Fig. 4a, *v*(*r*)=2 μm s^−1^, then linearly decays to *v*(*r*)=1 μm s^−1^ at *r*=50 μm and fades to zero at even longer distances. The optical forces induced by smooth and rough potentials are modelled by imposing an external force field acting on the particles **F**=[*F*_*x*_, *F*_*y*_]^34^: in the case of the smooth optical potential, the forces are calculated as the gradient of a 2D Gaussian potential; while in the case of the random potential, the forces are calculated as the gradient of a 2D speckle intensity pattern with a 2D Gaussian envelope and same average grain size as in the experiments^16^,^23^,^33^. Inertial effects can be neglected because of the very low Reynolds number regime of our system, while, when a displacement makes two particles overlap, the particles are separated by moving each one half the overlap distance along their centre-to-centre axis. The simulations are robust and the main observable result, that is, the transition from gathering to dispersal of active particles in an attractive potential, depends very little on the particular choice of the parameters (for example, absolute value and functional form of the velocity, and rotational diffusion), although the time dynamics for the two processes can be altered.

## Additional information

**How to cite this article:** Pinçe, E. *et al.* Disorder-mediated crowd control in an active matter system. *Nat. Commun.* 7:10907 doi: 10.1038/ncomms10907 (2016).

## Supplementary Material

Supplementary Information Supplementary Figures 1-3, Supplementary Table 1, Supplementary Notes 1-3 and Supplementary References.
