# Supplementary material for: Disorder-mediated crowd control in an active matter system
Source: Nat Commun. 2016 Mar 9;7:10907. doi: 10.1038/ncomms10907 (PMC4786875; doi:10.1038/ncomms10907)
Supplement: Supplementary Information — Supplementary Figures 1-3, Supplementary Table 1, Supplementary Notes 1-3 and Supplementary References. [file ncomms10907-s1.pdf]

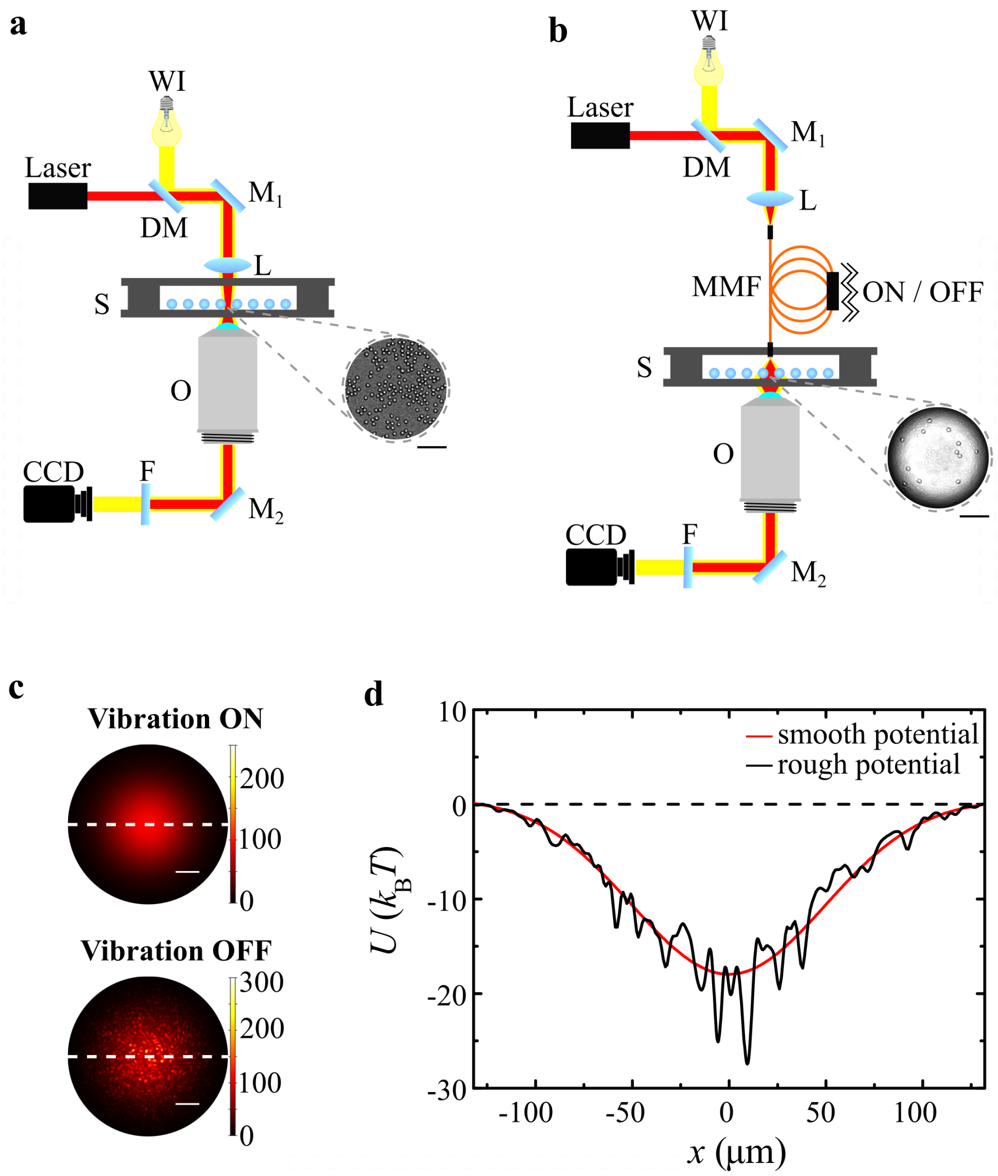

Supplementary Figure 1 | Schematics of the setup. Optical setups to generate (**a**) smooth Gaussian optical potentials and (**b**) optical potentials with a controllable degree of disorder: WI, white light lamp; DM, dichroic mirror; M_1 and M_2, mirrors; L, lens; S, sample; O, objective; F, filter; CCD, CCD camera; MMF, multimode optical fiber. (**c**) The fiber in **b** is attached to a mechanical oscillator whose vibration frequency can be modulated to change the roughness of the optical potential. (**d**) The profiles of the optical potentials along the white dashed lines in **c** both for smooth Gaussian illumination (red line) and disordered illumination (black line). All scale bars correspond to 40 μm.

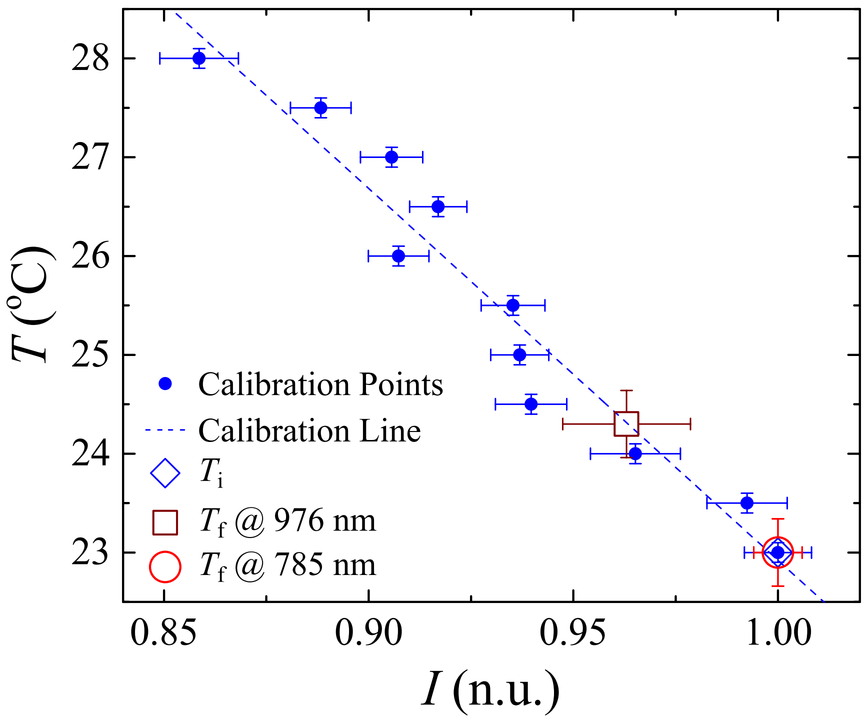

Supplementary Figure 2 | Temperature increase measurement. The normalized fluorescence intensity of Rhodamine B solutions in the sample chamber is a sensitive indicator of the sample solution temperature. In absence of laser illumination, the temperature of the sample cell is the room temperature (blue diamond, 23 °C). Under illumination with a laser at λ = 976 nm (P = 100 mW, w_0 = 47.8 ± 0.2 μm, brown square) the temperature increases by ΔT = 1.3 ± 0.3 K. Under illumination with a laser at λ = 785 nm (P = 100 mW, w_0 = 49.9 ± 0.2 μm, red circle) the temperature of the sample does not change appreciably. The dashed line shows the calibration curve that relates fluorescence intensity to temperature used to perform the measurements. This curve is the linear fitting line for the calibration data points (dots). Error bars represent one standard deviation around the mean values averaged over 6 measurements per data point.

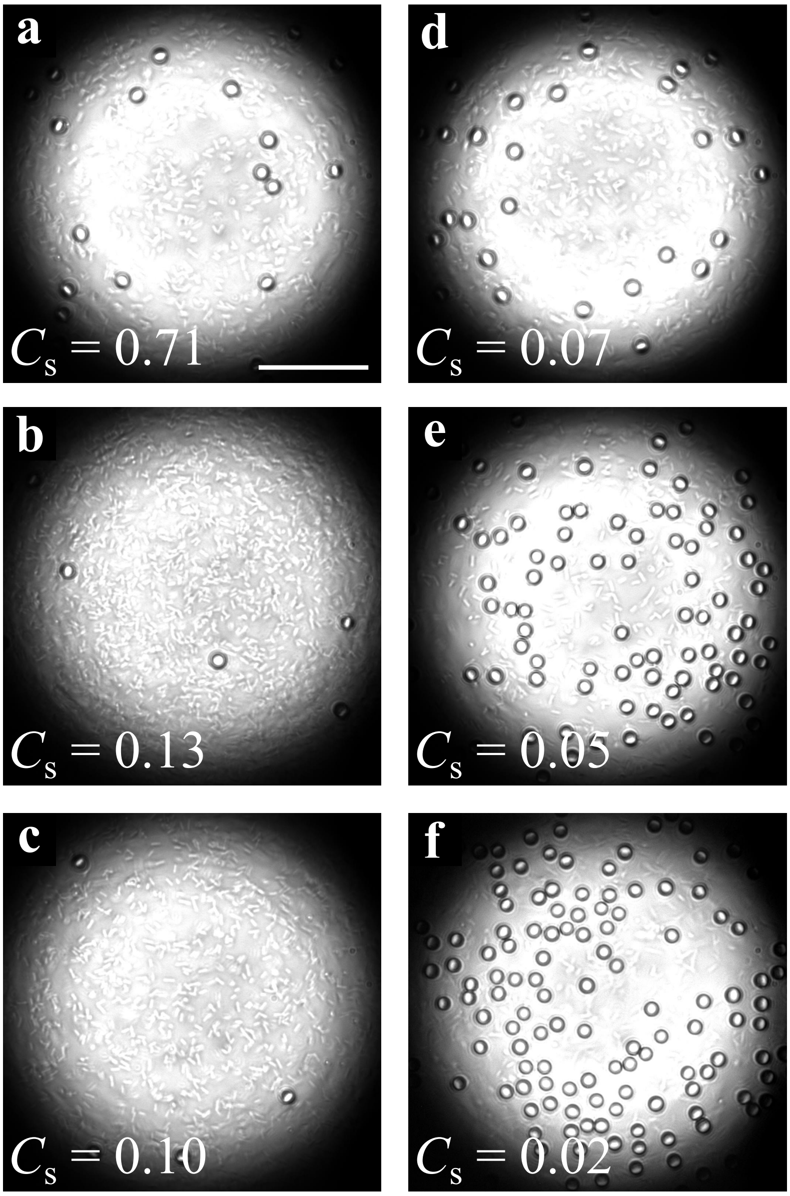

Supplementary Figure 3 | Final distribution of colloids for different speckle contrasts. Snapshots at t = 30 minutes of the distribution of colloids in an active bath under different optical potentials where the local roughness is continuously decreased from a high contrast speckle C_s = 0.71 (**a**) to an almost Gaussian distribution with very low speckle contrast C_s = 0.02 (**f**) through four intermediate values (**b**) C_s = 0.13, (**c**) C_s = 0.10, (**d**) C_s = 0.07, (**e**) C_s = 0.05. These snapshots show a non-monotone transition from dispersal (**a**) to gathering (**f**) of individuals in the central illuminated area. The scale bar corresponds to 40 μm. The complete time evolutions of the colloidal population are shown in Fig. 5**d**-**f** and Fig. 5**j**-**l**.

Optical Potential	Bath	r (μm)	v_r (μm s^{-1})
smooth	thermal	30	-0.37
smooth	active	30	-0.11
rough	thermal	30	-0.26
rough	active	30	+0.09

Supplementary Table 1 | Radial drift. Radial drift v_r at r = 30 μm for colloids in thermal and active baths under different potentials. The radial drift is positive only for colloids in an active bath moving on a rough potential (Supplementary Note 2).

Supplementary Note 1: Absence of convection and thermophoresis.

For our experimental conditions, we can exclude a convection-driven flow for the particles since the Rayleigh number

$$\mathrm{Ra} = \frac{g\alpha\Delta T l^3}{\upsilon\kappa} \approx 0.02 \ll 1700, \quad (1)$$

where 1700 represents the critical value above which the onset of convection occurs in the Bérnard configuration corresponding to two parallel horizontal boundaries separated by a distance $l = 100\ \mu\mathrm{m}$ and with a temperature difference $\Delta T = 1.2\ \mathrm{K}$; $g = 9.8\ \mathrm{m\ s^{-2}}$ is the gravitational acceleration, $\alpha = 2 \cdot 10^{-4}\ \mathrm{K^{-1}}$ is the thermal expansion coefficient of water, $\nu = 10^{-6}\ \mathrm{m^2\ s^{-1}}$ is its kinematic viscosity, and $\kappa = 0.14 \cdot 10^{-6}\ \mathrm{m^2\ s^{-1}}$ is its thermal diffusivity. Moreover, since silica particles have a positive Soret coefficient, thermophoresis can be excluded because, in absence of bacteria, they would otherwise be pushed away from the warmer central illuminated area towards the colder outside boundaries rather than gathering at its center.

Supplementary Note 2: Radial drift calculation.

The radial drift $v_\mathrm{r}(r)$ quantifies the average velocity at which the colloids move away from the center of the illuminated area along any radial direction, and it is defined as

$$v_\mathrm{r}(r) = \frac{1}{\Delta t}\langle r_{n+1} - r_n | r_n \cong r\rangle, \quad (2)$$

where r_n are discrete samples of the radial position of the colloidal particles calculated from the center of the optical beam and Δt is the time step between samples. A negative radial drift means the particles are gathering at the center, while a positive radial drift indicates that the particles are moving away from it. In a thermal bath, the effective radial drift of the colloidal particles is negative both in smooth and rough optical potentials; in an active bath, the radial drift is negative only in a smooth potential, while it is positive in a disordered potential, thus confirming the dispersal of colloids in this case (Supplementary Table 1).

Supplementary Note 3: Heating effects.

We measured the temperature increase over room temperature (fixed at 23 °C) at the center of the illuminated area (Supplementary Fig. 2) by fluorescence intensity measurements of Rhodamine B (Merck Millipore) using a photodiode [1]: in particular, our sample chamber was filled with a solution of rhodamine B (0.1 mM) prepared in carbonate buffer (0.02 M); all solutions were prepared using deionized water. The calibration curve in Supplementary Fig. 2 is obtained by linearly fitting the data points acquired by measuring the fluorescence intensity of the solution (excitation λ = 532 nm, P = 5 mW to avoid photobleaching) at different temperatures on a microscopy stage with temperature controlled at ± 0.05 K (using a heat bath). Under laser

illumination at λ = 976 nm (P = 100 mW, w_0 = 47.8 ± 0.2 μm, brown square), we measured a temperature increase of 1.3 ± 0.3 K. We remark that the absorption coefficient of water at λ = 785 nm is approximately twenty times smaller than at λ = 976 nm, thus the temperature variation will also be approximately twenty time smaller for the same power of the illumination, as confirmed from the measurements in Supplementary Fig. 2 where we did not observe any temperature increase at λ = 785 nm (P = 100 mW, w_0 = 49.9 ± 0.2 μm, red circle).

To gain insight on the temperature distribution beyond the spatial resolution of our measurements, for the case of light absorption from the motility buffer in our sample chamber (dimensions w_x = 1 mm, w_y = 1 mm, w_z = 0.1mm), we can calculate the temperature variation ΔT above room temperature due to laser illumination by numerically solving the steady-state Fourier's law of heat conduction [2]:

$$\nabla^2\big(\Delta T(\mathbf{r})\big) = -\frac{\mu(\lambda)}{C} I(\mathbf{r}), \tag{3}$$

where $I(\mathbf{r})$ is the intensity of the laser beam at point $\mathbf{r}$; $\mu(\lambda)$ is the absorption coefficient of the water in the buffer at a given wavelength; and C is its thermal conductivity. In our case, $I(\mathbf{r})$ is either given by a Gaussian beam or a speckle pattern with a Gaussian envelope, both propagating along z. For water, $\mu(\lambda)$ = 44.5 m^{-1} at λ = 976 nm, $\mu(\lambda)$ = 2.22 m^{-1} at λ = 785 nm and C = 0.60 Wm^{-1}K^{-1}. The intensity $I(\mathbf{r})$ is defined on a discretized lattice (spatial step: 1 μm) so that the total power at any *xy*-plane is equal to 100 mW (as the effect of absorption is negligible on these length scales). The temperature increases obtained from the numerical simulations shown in Fig. 2 are in good agreement with the measured values.

Supplementary References

1 Ross, D., Gaitan, M. & Locascio, L. E. Temperature measurement in microfluidic systems using a temperature-dependent fluorescent dye. *Anal. Chem.* **73**, 4117–4123 (2001).

2 Peterman, E. J. G., Gittes, F. & Schmidt, C. F. Laser-induced heating in optical traps. *Biophys. J.* **84**, 1308–1316 (2003).
